# Human linker histones: interplay between phosphorylation and O-β-GlcNAc to mediate chromatin structural modifications

**DOI:** 10.1186/1747-1028-6-15

**Published:** 2011-07-12

**Authors:** Waqar Ahmad, Khadija Shabbiri, Noreen Nazar, Shazia Nazar, Saba Qaiser, Mirza Abid Shabbir Mughal

**Affiliations:** 1 Centre of Excellence in Molecular Biology, University of the Punjab, Lahore, Pakistan; 2 Department of Chemistry, GC University, Lahore, Pakistan

## Abstract

Eukaryotic chromatin is a combination of DNA and histone proteins. It is established fact that epigenetic mechanisms are associated with DNA and histones. Initial studies emphasize on core histones association with DNA, however later studies prove the importance of linker histone H1 epigenetic. There are many types of linker histone H1 found in mammals. These subtypes are cell specific and their amount in different types of cells varies as the cell functions. Many types of post-translational modifications which occur on different residues in each subtype of linker histone H1 induce conformational changes and allow the different subtypes of linker histone H1 to interact with chromatin at different stages during cell cycle which results in the regulation of transcription and gene expression. Proposed *O*-glycosylation of linker histone H1 promotes condensation of chromatin while phosphorylation of linker histone H1 is known to activate transcription and gene regulation by decondensation of chromatin. Interplay between phosphorylation and *O-β*-GlcNAc modification on Ser and Thr residues in each subtype of linker histone H1 in *Homo sapiens *during cell cycle may result in diverse functional regulation of proteins. This *in silico *study describes the potential phosphorylation, o-glycosylation and their possible interplay sites on conserved Ser/Thr residues in various subtypes of linker histone H1 in *Homo sapiens*.

## Introduction

Eukaryotic genome is packaged into a structure known as chromatin. The basic structural unit of chromatin called as nucleosome is composed of DNA and proteins [1]. The major proteins involved in chromatin structure are histone proteins. Histone proteins are of five types: H1, H2A, H2B, H3 and H4 [2-4]. Histone H1 is known as linker histone while the other four histone proteins are collectively known as core histones. This DNA-protein complex is the tempelate for a number of essential cell processes including transcription recombination, repair and replication. Histone H1 is located on the linker DNA that goes between the nucleosomes in chromatin structure [5]. Linker DNA which is associated with linker histone H1 interconnects core particles, varies in length, depending on species and tissue [6]. Organization of DNA into nucleosomes by histone proteins and folding of nucleosomes into higher-order chromatin structure is generally believed to compact DNA and make it inaccessible to transcription factors [7]. Linker histones H1 are necessary for modulating chromatin structure and function at multiple levels [8].

Organisms contain a variety of subtypes of linker histone which exhibit significant sequence divergence and distinct patterns of expression differentiation and development [9]. The H1 linker histones are the most divergent group. Usually nine subtypes of linker histone H1 are present in mammals including H1.1, H1.2, H1.3, H1.4, H1.5, H1o, H1Foo, H1.t [10] and H1.x [11]. Linker histone sub-types are classified according to their tightly regulated expression pattern during embronyal development and cell differentiation [12]. All known sub-types of linker histone contain a common domain structure. Linker histones consist of a short *N*-terminal, a highly conserved central globular domain and a long *C*-terminal domain [13]. Somatic cells contain almost all sub-types of linker histone H1 [12]. *In vitro*, H1-containing chromatin shows strong inhibition of transcription [14] and transcriptionally active chromatin typically depleted in H1 compared with inactive chromatin [15]. Wisniewski *et al*. showed that many of the mapped modification sites which are thought to be involved in binding to nucleosomal DNA are located within the globular domain region of the different subtypes of the linker histone H1 [16]. H1 depletion results in a dramatic lengthening of chromosomes, which suggests an important role in mitotic chromosome condensation [17]. The presence of these large number of various H1 histone subtypes and their possible post-translational modifications, make it very clear that H1 histones play numerous structural and functional roles in chromatin [18]. Until now, no specific role for the various variants has been established but it is known that the mouse histone H1.2 binds preferentially to a regulatory sequence within a mouse H3.2 replication-dependent histone gene [19].

Post-translational modifications (PTMs) of linker histone H1 play very important role in regulation of chromatin structure, transcriptional regulation, gene activity [17] and controlling the accessibility of transcription factors to chromatin structure [20]. A working model of the cell cycle has slowly been constructed from the discovery of cyclins 22 years ago. This model is composed of protein phosphorylation, acetylation timed expression of cyclins, and well orchestrated cell division. Nevertheless, a detailed mechanism of the cell cycle is still incomplete [21-23]. Transcriptional activation of genes starts with the dissociation of linker histone H1 from linker DNA [24]. Phosphorylation of linker histone is required for efficient cell cycle progression by enzyme CDK2 [25]. These kinases requires a consensus sequence (S/T)PXZ or (S/T)P*X*K for phosphorylation (where X is any amino acid and Z is a basic amino acid) and this consensus sequence is found in many linker histone H1 variants which become phosphorylated [26]. It is found that PKC is also involved in phosphorylation of linker histone variants during regulation of gene expression in cell cycle [27]. Phosphorylation of linker histone regulates transcription and gene expression by reducing the electrostatic binding of linker histone to DNA in chromatin [28]. *In vivo *phosphorylation of the linker histone tails influence both the binding to mononucleosomes and the aggregation of polynucleosomes [29]. The phosphorylation of linker histones at their *N *and *C*-terminal tails during the cell cycle influence its functions for enhancing decondensation which in turn regulate transcription and gene expression. This phosphorylation and dephosphorylation is a common regulatory mechanism for protein functions [30].

*O*-Glycosylation is also very important PTM of proteins. During *O*-Glycosylation one molecule of N-acetylglucosamine (*O-β*-GlcNAc) is introduced on Ser or Thr residue by enzyme *O*-GlcNAc transferases (OGT). Addition of *O-β*-GlcNAc can inhibit phosphorylation on Ser or Thr residue and is reciprocal with phosphorylation on some well studied proteins, such as RNA polymerase II, estrogen receptor, and the c-Myc proto-oncogene product [31-34]. These studies suggest that O-GlcNAc may function as a global regulator of cell growth and division. Deletion of OGT in mouse embryonic fibroblasts is associated with delayed growth, increased expression of the cyclin inhibitor p27, and death. A reduction in O-GlcNAc levels results in cell growth defects, by the lowering UDP-GlcNAc levels to 5% of normal [35,36]. Studies in *Xenopus *demonstrated maturation defects in oocytes when microinjected with galactosyltransferase which prevents O-GlcNAc removal. Meanwhile incubation of *Xenopus *oocytes with the O-GlcNAcase inhibitor PUGNAc altered progression of oocytes through progesterone-mediated maturation [37-40].

In 1994, Kim *et al*. first time observed the o-GlcNAc modification in mouse linker histone H1. They also observed same PTM on core histones [41]. In 2005, Slawson *et al*. showed that increased O-GlcNAc resulted in growth defects linked to delay in G2/M progression, altered mitotic phosphorylation, and cyclin expression. Over expression of O-GlcNAcase, the enzyme that removes O-GlcNAc, induces amitotic exit phenotype accompanied by a delay in mitotic phosphorylation, altered cyclin expression, and pronounced disruption in nuclear organization. Overexpression of the O-GlcNAc transferase, the enzyme that adds O-GlcNAc, results in a polyploid phenotype with faulty cytokinesis. Notably, O-GlcNAc transferase is concentrated at the mitotic spindle and mid body at M phase. These data suggest that dynamic O-GlcNAc processing is a pivotal regulatory component of the cell cycle, controlling cell cycle progression by regulating mitotic phosphorylation, cyclin expression, and cell division [42]. On the basis of above observations, Kaleem et al (2008) used bioinformatics tools to predict o-glycosylation on human core histone H3, even though there was no experimental proof of that PTM on histone H3 [43].

Interplay between *O-β*-GlcNAc modification and phosphorylation on the same amino acid residues has been observed in several nuclear and cytoplasmic proteins [44]. These PTMs are dynamic and result in temporary conformational changes and regulate many functions of the proteins. The alternation of these two modifications on the same or neighboring residue may modulate the specific function of the proteins either by enhancing or inhibiting the functional capacity. Residues where *O-β*-GlcNAc and phosphorylation compete for each other are known as Yin Yang sites [45]. These Yin Yang sites can be predicted and analyzed using various computer-assisted neural network-based programs, which can help us to determine proteins regulatory functions by accessing their modification potentials.

Although a yin/yang relationship between phosphorylation and *O*-GlcNAcylation on histone H3 has been proposed; the direct evidence for *O*-glycosylation of histones was never been described. Recent studies by Sakabe *et al *(2010-2011) first time proved the O-GlcNAc modification on histones and also mapped glycosylation sites with specific immunological, enzymatic and mass spectrometric techniques. They also insist to include O-GlcNAc modification as part of histone code. They showed that histone O-GlcNAcylation increases with heat shock and this increase is concomitant with DNA condensation [46,47]. The present work describe potential phosphorylation, *O*-Glycosylation and their possible interplay sites which influence condensation, decondensation and transcriptional and gene regulation during cell cycle in various subtypes of linker histone H1.

## Materials and Methods

The sequences of different types of linker histone H1 of many species mostly mammals have been described by many workers [10,11,16]. The sequence data used for predicting phosphorylation and glycosylation sites for different subtypes of linker histone H1 of *human *was retrieved from the SWISS-PROT [48] sequence database. The primary accession numbers for each subtype of linker histone in human are Q02539 (H1.1), P16403 (H1.2), P16402 (H1.3), P10412 (H1.4), P16401 (H1.5), Q81ZA3 (H1oo), P22492 (H1.T), P07305 (H1.0) and Q 92522 (H1.X). BLAST search was made using the NCBI database of non-redundant sequences [49]. The search was done for all organisms' sequences with expect value set to 10 using blosum 62 matrix and low complexity filter selecting nr database. Hits with highest bits score and zero expect value were selected. The four to five sequences of each subtype of linker histone H1 from different selected species were selected to find out conserved residues in *Homo sapiens *linker histone H1. All selected sequences were multiple aligned using CLUSTALW [50]. All the sequences of subtypes of linker histone H1 present in *Homo sapiens *were aligned to get the conservation status of subtypes.

### Post-translational modifications prediction methods

Phosphorylation sites on Ser, Thr and Tyr residues were predicted by using NetPhos 2.0 (http://cbs.dtu.dk/services/NetPhos/) server [51]. NetPhos 2.0 is a neural network-based method for the prediction of potential phosphorylation sites.

NetPhosK 1.0 server (http://cbs.dtu.dk/services/NetPhosK) [52] was used to predict kinase specific phosphorylation sites in human histone H1.

Phospho.ELM database (http://phospho.elm.eu.org/) was used for the determination of the experimentally verified phosphorylation sites [53] present on various linker histone H1 subtypes in different species. The Phospho.ELM database contains a collection of experimentally verified Ser, Thr and Tyr sites in eukaryotic proteins.

To predict potential *O-β*-GlcNAc modification sites, YinOYang 1.2 (http://www.cbs.dtu.dk/services/YinOYang/) was used. This method is also capable of predicting the potential phosphorylation sites as well and hence predicting the Yin Yang sites [54-56].

### Neural networks-based prediction methods

Artificial neural networks based methods have been extensively used in biological sequence analysis and predicting the potentials for modifications [57]. The methods developed using machine learning approach includes memorizing the neural networks with the sequence environment windows of phosphorylated/glycosylated and non-phosphorylated/non-glycosylated sites. During this learning process the input data of phosphorylated/glycosylated and non-phosphorylated/non-glycosylated sites is presented to neural networks in the form of binary codes of 21 digits. A threshold value in form of bits is set for positive hit and zero for negative hits. The learning process and performance is checked with the data reserved for cross validation using statistical equations. During learning, the error is computed and weights given to each neuron are set to get the maximum correct predictions.

## Results

### Allignment of sequences for determination of conserved status of Ser/Thr residues within different linker histone subtypes

**Figure 1:**
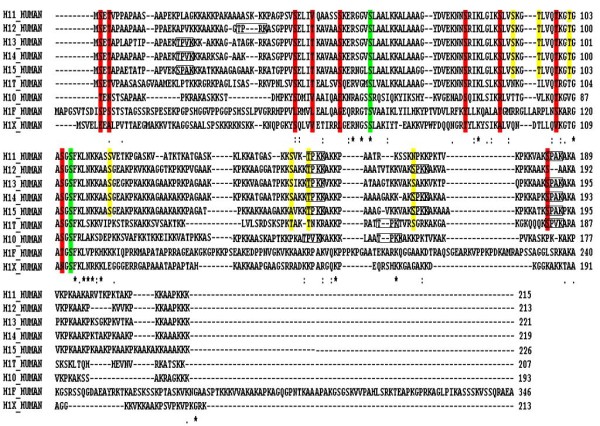
**Sequence alignment of different subtypes of linker histone H1 present in Homo sapiens**. The residues highlighted in red show conserved and conserved substitution regions in Ser and Thr residues, while the regions highlighted in yellow show that Ser and Thr residues which are conserved in maximum subtypes but not present in all of the subtypes in linker histone H1. The consensus sequences (motifs) for phosphorylation are shown in square lines.

Each human linker histone subtype was aligned with other species. Conserved and conserved substituted Ser and Thr residues within each subtype were determined (Data not shown). These nine subtypes were also aligned with each other to find conserved residues within subtypes (Figure 1).

### Prediction of phosphorylated S/T residues with motif

(S/T)PXZ and (S/T)PXK motifs were searched for each linker histone H1 subtypes. Sequences within boxes showed the specific motifs (Figure 1). These residues are given in Table 1.

**Table 1:** Phosphorylation and *O-β*-GlcNAc site map of *Homo sapiens*

Substrate		Phosphorylation Sites by NetPhos	Experimentally known	Predicted by Motif	Yin Yang sites	Conserved	Conserved sub
**H1.1**	SER	33, 41, 51, 52, 53, 91, 106, 114, 115, 123, 135, 145, 148, 164, 165	1, 35, 103, 183	183	33, 52, 53, 114, 164, 165	41, 43, 51, 53, 60, 106, 183	1, 48, 52, 91, 103
	THR	94, 151, 161, 173, 199, 203	151	151	161, 173, 199, 203	94	101, 151, 11, 164, 203
**H1.2**	SER	35, 50, 54, 104, 112, 149, 172, 187	1, 172	172	30, 50, 187	1, 40, 58, 77, 102, 104, 172, 187	35, 85, 88, 112
	THR	30, 91, 145, 153, 166	30	30, 145, 153	145, 166	44, 91, 95, 98	3, 153
**H1.3**	SER	36,51, 55, 104, 113, 150, 173, 188, 204	188	173, 188	35, 51, 188, 204	36, 41, 51, 58, 79, 89, 102, 104, 188	1, 86
	THR	18, 92, 146, 154, 167, 179	18	18, 146, 154	146	3, 45, 92, 96, 99	154
**H1.4**	SER	26, 35, 50, 54, 103, 112, 150, 171, 186	35, 171, 186	171, 186	35, 50, 186	1, 35, 40, 50, 54, 57, 78, 85, 88, 101, 103, 112, 150, 171, 186	172, 188
	THR	17, 91, 145, 153, 202	17	17, 145, 153	17, 145, 202	3, 17, 91, 95, 98, 145	141, 153, 202
**H1.5**	SER	17, 43, 53, 106, 115, 172, 188	17, 172, 188	17, 172	17, 43, 53	1, 43, 60, 80, 104, 106, 115	17, 53, 88, 91, 172
	THR	10, 24, 38, 94, 137, 154	137,154	10, 137, 154	10, 38	38	3, 8, 47, 98, 101, 154
**H1.0**	SER	6, 18, 21, 44, 48, 65, 70, 97, 103, 123, 130, 185	123		6, 21, 44, 97, 103, 123, 130	4, 6, 21, 28, 44, 45, 55, 65, 70, 89, 91, 103, 130, 170, 184, 185	18, 97, 115
	THR	109, 118, 134, 140, 152, 161		118, 140, 152	134, 161	1, 5, 22, 76, 77, 83, 109, 118, 123, 134, 140, 152	161
**H1.T**	SER	8, 42, 52, 54, 86, 107, 111, 118, 126, 128, 137, 140, 142, 165, 180, 187, 204	177	142, 180	8, 54, 118, 180, 204	1,42, 44, 52, 54,61,81, 105,107,140, 142,165, 180	8, 35, 126, 128, 137, 187, 189, 204
	THR	131, 148, 158, 159, 162, 203	158, 159		148, 158, 159, 162, 203	3, 21, 99, 102, 148, 158	10, 48, 131, 145, 203
**H1oo**	SER	8, 11, 13, 14, 16, 20, 21, 23, 26, 32, 42, 73, 161, 211, 229, 230, 235, 243, 245, 246, 260, 262, 263, 276, 336, 337, 340, 341		276	8, 13, 14, 16, 26, 73, 229, 262, 336, 337, 340, 341	5, 8, 12, 13, 20, 67, 110, 118, 221, 236	7, 122, 219, 231, 241, 249
	THR	72, 194, 256, 278, 319			256, 319	66, 81, 97, 116, 231	19, 209
**H1.X**	SER	31, 33, 39, 92, 113, 154, 171	31, 33		33	49, 65, 66, 92, 113,	27, 31, 133
	THR	55				101	12, 13, 55, 87

### Acquiring of experimentally verified S/T/Y residues

Data for experimentally confirmed S/T/Y residues was obtained from Phospho ELM and UniprotKB (http://www.uniprot.org) is given in Table 1. All histone H1 subtypes phosphorylated during cell cycle except H1oo.

### Prediction of Phosphorylation Sites

**Figure 2:**
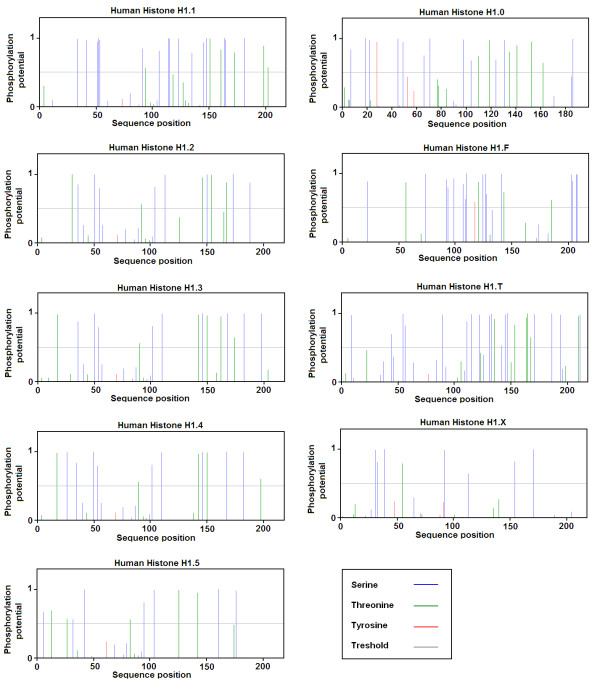
**Graphical presentation of potential for phosphate modification at Ser, Thr and Tyr residues in different subtypes of linker histone H1 in Homo sapiens**. Here blue vertical line show the phosphorylation potential of Ser, green vertical lines show the phosphorylation potential of Thr residues, redlines show phosphorylation potential of Tyr residues, and gray horizontal lines show threshold for modification potential in each subtype of linker histone H1.

NetPhos 2.0 server was used for the prediction potential for phosphorylation of possible Ser and Thr residues among all known subtypes of linker histone H1. All the subtypes of linker histone H1 showed high potential for phosphorylation as shown in Figure 2. The predicted Ser and Thr residues are shown in Table 1.

### Prediction of Kinases involved in Phosphorylation

Different kinases are involved in phosphorylation of Ser and Thr residues of linker histone H1 subtypes. Almost each kinase predicted is involved in phosphorylation of two or more residues. The predicted kinases involved in phosphorylation by NetPhos K 1.0 are shown in Table 2.

**Table 2:** Protein kinases invoved in phosphorylation of different subtypes of linker histone H1 in *Homo sapiens*

Histone H1 Sub-types		Enzymes for Phosphorylation HUMAN							
		
		PKC	PKA	CDC2	CDK5	GSK3	P38 MAPK	RSK	PKG
**H1.1**	SER	33, 52, 104, 106, 115, 123, 145, 148, 164	41,123,	51, 52, 53	182	182		164, 165	11, 165
	THR	3, 94, 101, 118, 127, 132, 142, 151, 161, 173, 199, 203			151		151		
**H1.2**	SER	50, 57, 85, 101 103, 112, 149, 172, 187	35	50					37, 149
	THR	30, 91, 98, 125 153, 164, 166,			145, 153,		30		
**H1.3**	SER	51, 58, 86, 102 104, 113, 150, 173, 188, 204	36,	51,	188,				36,
	THR	29, 92, 99, 154 167, 210		9,	17,146, 154,	146	17, 146, 179,		210
**H1.4**	SER	26, 50, 57, 85 101, 103, 112, 149, 171	26, 35,	50,	187	187	187	171	26, 35, 149,
	THR	91, 98, 141, 153, 202			17, 145, 153,		17,		
**H1.5**	SER	53, 88, 104, 106, 115, 172, 188,	60,		17, 172, 188	172	188		
	THR	24, 38, 94, 101 137, 154, 186,	38,		137, 154,		10, 137,		8, 38, 154,
**H1.O**	SER	18, 44, 45, 55, 70, 91, 103, 123, 130, 170, 184, 185	18, 28, 44,	4, 6, 21,					18, 185
	THR	22, 76, 109, 118, 134, 152, 161	89,				140		22, 109
**H1.F**	SER	20, 73, 124, 211, 235, 243, 256, 260, 263, 268, 276, 306, 335, 336, 337, 341	42,	13, 14, 16, 21, 45,	11,		23, 276	73,	207, 243, 256
	THR	17, 103, 194, 266, 278, 297	72,						266,
**H1.T**	SER	35, 86, 89,105, 107, 111, 118, 121, 126, 128 137, 165, 187, 189, 204	42, 61, 86, 187	1, 33, 35, 44, 54, 111, 180	180		180	187	
	THR	102, 119, 131, 144, 148, 158, 162, 203			159				203
**H1.X**	SER	27, 33, 39, 92, 113, 154, 204	39, 49, 66,	65,	31	31		39	39, 204
	THR	87, 135, 140		135					189

### Prediction of O-Linked Glycosylation Sites

**Figure 3:**
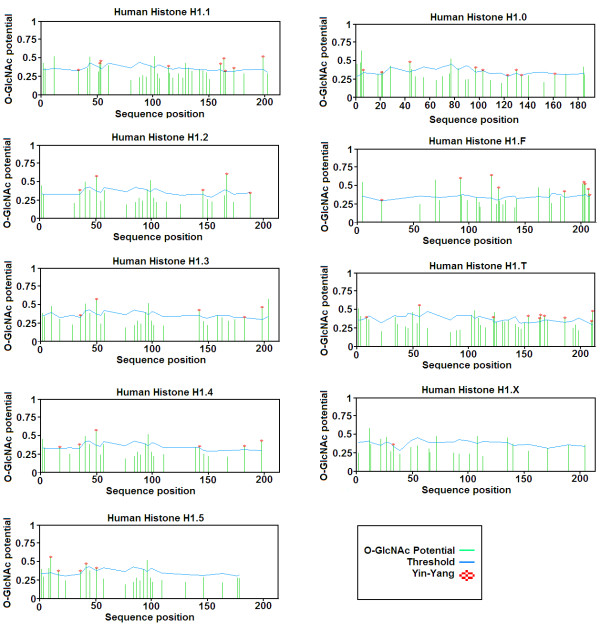
**Graphical representation of potential for *O-β*-GlcNAc modification in Ser and Thr residues in the different subtypes of linker histone H1 in Homo sapiens**. Green vertical lines show the potential of Ser/Thr residues for *O-β*-GlcNAc modification and light blue horizontal wavy lines show threshold for modification potential.

Prediction results for *O*-linked glycosylation sites showed that all subtypes of linker histone H1 have very high potential for *O-β*-GlcNAc modification Table 3. There are many predicted Yin Yang sites in each subtype of linker histone which are shown by an asterisk as shown in Figure 3.

**Table 3:** Proposed Ser/Thr residues for interplay of phosphorylation and *O-β*-GlcNAc modification in different subtypes of linker histone H1 in *Homo sapiens*

SUBSTRATE		Proposed Yin Yang sites	Proposed Fn-Yin Yang sites
**H1.1**	SER	103, 183	41, 51, 91, 104, 106, 182
	THR	203	94, 203
**H1.2**	SER	187	-
	THR	-	-
**H1.3**	SER	188	104
	THR	146	92, 154
**H1.4**	SER	35, 186	54, 103, 112, 171
	THR	17, 45, 202	91, 153
**H1.5**	SER	17	106, 115, 172
	THR	-	-
**H1.0**	SER	21, 44, 97, 103, 123, 130	-
	THR	134, 161	-
**H1.T**	SER	54, 180, 204	42, 52, 107, 126, 128, 137, 140, 165, 187
	THR	148, 158, 203	31
**H1oo**	SER	8, 13	-
	THR	-	-
**H1.X**	SER	-	
	THR	-	-

### Identification of False-Negative Sites

The Ser and Thr residues which were not predicted to be *O-β*-GlcNAc modified but have very high potential for phosphorylation and very close to threshold value are known as false-negative sites (FN-sites). All the Ser and Thr residues which were predicted false-negatively with high conservation status and phosphorylation potential among different subtypes of linker histone H1 are given in Table 3.

### Possible proposed YinYang sites within different subtypes of linker histone H1

The possible proposed Yin Yang sites for the interplay of phosphorylation and *O-β*-GlcNAc modification are given in Table 3. These Yin Yang sites are proposed on the basis of conservation status of Ser/Thr residues in each subtype of linker histone H1. The Ser/Thr residues are also proposed for the possible interplay of phosphorylation and *O-β*-GlcNAc modification on the basis of their similarity with other species. These Ser/Thr residues which are predicted "by similarity" are not yet experimentally known in *Homo sapiens *but these are known in other species of vertebrates.

## Discussion

Human linker histones have more than eight sub-types, all consisting of a highly conserved globular domain and less conserved *N*- and *C*-terminal tails. The sequence of terminal tails of different subtypes of linker histone H1 within a species is much less conserved but the sequence of terminal tails of a specific subtype is well conserved among different species [58]. In addition to heterogeneity of their primary structures, the histone tails are also post-translationally modified under various biological conditions [59]. The proportion of linker histone H1 subtypes varies in a tissue- and species-species manner [60], and the expression of each subtype varies throughout development and differentiation [61]. Studies of the structure of different subtypes of linker histone H1 and their interaction with the nucleosome and their roles in controlling gene activity indicate that linker histones have both an essential architectural function and an important task in regulating transcription [2]. The precise functions and modifications of linker histones are not yet fully understood, but it is known that different linker histone variants are preferentially localized to particular chromosomal domains. The sequences within the globular domain of linker histone H1 are thought to be responsible for the differential effect of overproduction of different linker histone variants on gene expression [62], while the *N*- and *C*-terminal domains of linker histone H1 are responsible for the condensation of chromatin [63]. The *N*-terminal of linker histone H1 binds with linker DNA [64] and C-terminal of linker histone H1 has binding affinity with core histones [58]. Different linker histone H1 subtypes have different chromatin condensing abilities [65]. All linker histone H1 subtypes differ not only in primary sequence but also in turnover rate, timing of synthesis during development and extent of phosphorylation and they also have the potential to add a great deal of flexibility to chromatin structure and transcriptional activation [66]. Linker histone H1 is required for longitudinal compaction of replicated chromosome. Enrichment of linker histone H1 onto chromatin required passage through interphase, when DNA replication takes place. Thus, linker histone H1 contributes to chromosome condensation in vertebrates [67]. In mouse depletion of linker histone H1 caused chromatin structure changes which include decreased global nucleosome spacing, reduced chromatin compaction and decreased in certain histone modifications like methylation [68]. *In vitro *experiments showed that linker histone H1 represses transcriptional promoters and factors by condensing the chromatin material [69] but *in vivo *studies showed that linker histone H1 does not function as a global transcriptional repressor, but instead participates in complexes that either activate or repress specific genes [70]. Differences between linker histone H1 subtypes for both binding and the capacity to aggregate polynucleosome into condensed structure implies functional differences between the different linker histone H1 subtypes during cell cycle and development of organism [71]. Sub-fractions of H1 histones differ in their effectiveness in condensing DNA fibers into ordered aggregates. Furthermore, each of linker histone H1 variant has differences in their binding capacity with DNA [72].

Hale *et al*. showed that phosphorylation of linker histone H1 provides a signal for the disassembly of higher order chromatin structure during cell cycle [73]. Linker histone H1 phosphorylated in a cell-cycle dependent manner, in G_1 _phase levels of H1 phosphorylation are usually lowest and then rise continuously during S and G_2 _phase. The M-phase where chromatin is highly condensed shows the maximum no. of phosphorylated sites [74]. The phosphorylation of linker histone H1 subtypes occurs on specific Ser and Thr residues during cell cycle in the presence of different protein kinases [75]. Interphase phosphorylation occurs mainly on Ser residues while during mitosis, Thr phosphorylation takes place [76]. The *C*-terminal domain of linker histone H1 not only makes up half of the linker histone molecule, but also has the abundant lysine/arginine residues and (S/T)P*X*K consensus sequences (phosphorylation motifs) [77]. The relative contributions of linker histone H1 binding amino acids and the (S/T)PXZ or (S/T)P*X*K motifs are examined. The presence of (S/T)P*X*K phosphorylation sites in histone H1.4 and H1.5 suggest that these DNA-binding motifs have greater influence on the binding affinities. The short *C*- terminal domain of linker histone H1.5 to the length of histone H1.2 results in a significant reduction in the binding of the H1.5 protein which demonstrates that the (S/T)P*X*K motifs are not the sole determinants of the affinity of histone H1 binding [78,79]. It is also very interesting to know that phosphorylation of linker histone also found in *N*-terminal regions where no (S/T)P*X*K consensus sequence found and so there is no absolute cell cycle specific site for phosphorylation [80]. Linker histone phosphorylation mainly depends upon their specific subtypes which occur during cell cycle at different residues. Linker histone H1.5 phosphorylated in both the *C*- and *N*-terminal regions while linker histone H1.2, H1.3 and H1.4 exclusively phosphorylated in the *C*-terminal regions [81].

Linker histones not only regulate gene expression and transcription but also have roles in ageing, DNA repair and apoptosis which suggest their importance in maintaining chromatin and genomic integrity [82]. These regulations are in response to changes in the ionic environment by electrostatic interactions between DNA, histone proteins, and free ions [6]. Decondensation of chromatin mediated through phosphorylation of linker histone that weakens the electrostatic interactions between the negatively charged DNA and positively charged *C*-terminal tails of linker histone subtypes and vice versa [83]. During mitosis linker histone H1.1 phosphorylated on two residues Thr-152 and Ser-182 [79], histone H1.2 phosphorylate on Ser-172, histone H1.3 phosphorylate on Ser-188, histone H1.4 phosphorylate on three residues including two Ser residues 171 and 186, and one Thr residue 145 while linker histone H1.5 phosphorylate on four residues, two Ser 17 and 172, and two Thr 137 and 154 [73]. Linker histone H1.T phosphorylates on three residues Ser-177, Thr-158 and 159 while H1.X also phosphorylates three residues Ser-2, 31 and 33 [83]. There is no experimental data available about the phosphorylated sites of other two remaining linker histone subtypes H1.F and H1.0 in mammals. It is found that during interphase, phosphorylation of Ser residues occurs while during mitosis Thr residues are phosphorylated. This shows the dual effect of linker histones phosphorylation during cell cycle; firstly during interphase the phosphorylation of Ser residues of all subtypes of linker histone H1 promotes DNA replication, transcription and gene regulation and then during mitosis phosphorylation of Thr residues of linker histone H1.4, H1.5 and H1.T may be required for recruiting proteins that are involved in condensation mechanism by unknown mechanism [84].

Our results of NetPhos K 1.0 for the prediction of phosphorylation potential of all Ser and Thr residues (which are experimentally known and described above and also involved in phosphorylation in different subtypes of linker histone H1) showed that these residues are phosphorylated by different kinases during cell cycle as shown in Table 2. These experimentally verified residues are conserved in all subtypes of linker histones in mammals and we can conclude that these phosphorylated sites can be present on linker histones of other mammals "by similarity" where these phosphorylation sites are not yet experimentally known. *O-β*-GlcNAc modification can occur on these Ser and Thr residues where kinases are involved in phosphorylation as it is well known that kinases and OGT can compete for same site modification [85]. This shows a possibility for interplay between phosphorylation and OGT on these residues. YinOYang 1.2 prediction results had shown that all subtypes of linker histone H1 of mouse have high potential for *O-*linked glycosylation (Figure 3). The proteins modified by *O-β*-GlcNAc are more concentrated on condensed chromatin as compared with transcriptionally active regions [86] thus the *O-β*-GlcNAc modification acts in a reciprocal manner to phosphorylation. Chromatin and several transcription factors are also found to be modified by OGT [87].

The Ser and Thr residues of linker histone H1 which are know to be experimentally phosphorylated and also showed positive potential for *O-β*-GlcNAc modification are Ser-188 of H1.3, Ser-186 and Thr-145 of H1.4, Ser-17 of H1.5 and Ser-177 of linker histone H1.T. NetPhos 2.0 prediction results showed that there are many Ser and Thr residues which are not yet experimentally verified but have high potential for phosphorylation, same as; YinOYang 1.2 also predicted such type of residues to have high potential for *O-β*-GlcNAc modification (Table 1). These predicted sites can also be phosphorylated by different kinases (Table 2) and act as possible Yin Yang sites for *O-β*-GlcNAc modification (Table 3). The remaining Ser and Thr residues of linker histone subtypes which are conserved in different species and either known or predicted to be phosphorylated, showed negative potential for *O-β*-GlcNAc modification but are very close to threshold value are known as false-negative Yin Yang (FN-Yin Yang) sites (Table 3). These conserved sites can be accessed by different kinases so that these sites have also strong possibility for OGT access and thus can also act as source of interplay for phosphorylation and *O-β*-GlcNAc [54-56]. The binding of DNA with nucleosome can be increased with the mutation of Ser and Thr phosphorylation sites to alanine residues at different subtypes of linker histone H1 [22]. This phenomenon has showed that these Ser and Thr residues are involved in transcription and gene regulation during cell cycle through interplay of phosphorylation and *O-β*-GlcNAc modification.

The above discussion reveals that all the conserved phosphorylated residues which show positive potential for *O-β*-GlcNAc modification or predicted as FN-Yin Yang sites as shown in Table3 may involved in modulating the functions through interplay between phosphorylation and *O-β*-GlcNAc modification among different subtypes of linker histone H1. These linker histone H1 subtypes phosphorylated on specific Ser residues at *N*-terminal region; enhance the process of DNA replication, transcription and gene regulation by decondensation of chromatin material during interphase. We propose that this decondensation process can be blocked by *O-β*-GlcNAc modification on these specific Ser residues which may result in chromatin condensation and repress transcription of DNA. Secondly the interplay between phosphorylation and *O-β*-GlcNAc modification on Thr residues during mitosis may activate proteins which are involved in condensation mechanism. Thus we can conclude that phosphorylation in different subtypes of linker histone H1 on proposed Ser/Thr residues is involved in decondensation of chromatin structure which leads to transcription regulation and gene expression, whereas the *O-β*-GlcNAc modification occurring on the same Ser/Thr residues may involved in condensation of chromatin. As histone O-GlcNAcylation is concomitant with DNA condensation, hyperthermia has been shown to sensitize tumor cells to radiotherapy. Although the mechanism for this sensitization has not been elucidated, it has been suggested that prior treatment with heat affects the cellular response to DNA damage induced by ionizing radiation and changes in histone O-GlcNAcylation might be another potential mechanism for radio-sensitization [47].

## Abbreviations

PTMs: post-translational modifications; Ser: Serine; Thr: Threonine; *O-β*-GlcNAc: N-acetylglucosamine; OGT: O-GlcNAc transferases; PUGNAc: *O*-2-acetamide-2-deoxy-D-glucopyranosylideneamino-*N*-phenylcarbamate.

## Competing interests

All authors have no any kind of institutional or financial competing interests.

## Authors' contributions

NN, SN, SQ and MASM collected and analyzed data. WA and KS design the study and wrote the manuscript. All authors read and confirmed the final manuscript.

## Authors' information

Ahmad W (M Phil Chemistry) is Research Officer at CEMB, University of the Punjab, Shabbiri K (M Phil Chemistry) and Qaiser S are Lecturers at GC University Lahore. Mughal MAS (MPhil Chemistry) is teaching assistant at GC University Lahore while Nazar N and Nazar S are BSc (Hons) students at GC University, Lahore.
